# Parietal Meningocele Under the Scalp of a Fetus Diagnosed Based on Volume Contrast Imaging of Prenatal Three-Dimensional Ultrasound Data

**DOI:** 10.1155/crnm/7401673

**Published:** 2025-01-08

**Authors:** Akihiro Hasegawa, Masami Kono, Tokumasa Suemitsu, Yuki Ito, Tatsuya Hirotsu, Yuichiro Nonaka, Osamu Samura, Aikou Okamoto

**Affiliations:** ^1^Department of Obstetrics and Gynecology, The Jikei University School of Medicine, Tokyo, Japan; ^2^Department of Neurosurgery, The Jikei University School of Medicine, Tokyo, Japan

**Keywords:** bone defect, meningocele, prenatal diagnosis, three-dimensional ultrasound, volume contrast imaging

## Abstract

Determining the differential diagnosis of small scalp cysts identified on a fetus is difficult. In particular, many physicians have difficulty differentiating small meningoceles from small scalp cysts during the prenatal period. Volume contrast imaging increases contrast between tissues, thereby allowing an enhanced view of target structures. A 15 × 5 mm scalp cyst was identified on a fetus during a prenatal ultrasonography examination performed at 20 weeks of gestation. The cyst was not connected to the blood flow, and did not include the tissue of the brain parenchyma. Ventriculomegaly and other structural abnormalities were not observed. Based on these findings, we suspected a sinus pericranii or fetal epidermal cyst. The size of the fetal scalp cyst was stable, and the growth of the fetus remained normal until birth. The diagnosis of a small meningocele was confirmed postnatally, based on the results of a magnetic resonance imaging examination. Postnatal evaluation of offline volume contrast imaging of prenatal three-dimensional ultrasound data at 22 weeks of gestation revealed a skull bone defect beneath the cyst. Volume contrast imaging can facilitate the prenatal diagnosis of small meningoceles by detecting bone defects on the fetal head.

## 1. Introduction

A small cyst under the scalp of a fetus is an ultrasonographic finding with a challenging differential diagnosis that can include a cephalocele, meningocele (MC), epidermal scalp edema, sinus pericranii, teratoma, lipoma, and hemangioma [1–9]. Among these, MC has the most important effect on both fetuses and parents; as such, accurate diagnosis is required for fetuses with small scalp cysts. Further, approximately 80% of MC cases result in pregnancy termination or intrauterine fetal demise [4, 10]. Ultrasonography and magnetic resonance imaging (MRI) are commonly used to prenatally diagnose fetal MC. Medium and large defects of the skull bone are generally easy to detect, and are usually accompanied by ventriculomegaly and hydrocephalus [10]. However, the prenatal diagnosis of a small MC is challenging because a clear border between the meninges and skull bone is often difficult to detect using two-dimensional ultrasonography because of the small size of the MCs, fetal head angles, fetal positions, ultrasound artifacts, and acoustic shadows [1, 5]. Three-dimensional ultrasonography and three-dimensional power Doppler imaging may aid in the diagnosis of small MCs; however, acoustic shadows and ultrasound artifacts create the appearance of cleavage between skull bones or suture lines, which can mimic a true head bone defect [1, 2]. Volume contrast imaging (VCI) is an ultrasonographic approach that utilizes three-dimensional volume data to display a three-dimensional structure parallel to the probe. VCI reduces speckle noise and improves contrast resolution, allowing better assessments of the size, margin, and internal aspects of structures and tissues [11]. VCI has been used in the prenatal evaluation of the bones of the extremities, long bones, and abnormalities of the cerebellar vermis of the fetus [12, 13]. Herein, we present a case of a MC observed as a bone defect on the fetal head that was diagnosed using VCI.

## 2. Case Presentation

**Figure 1:**
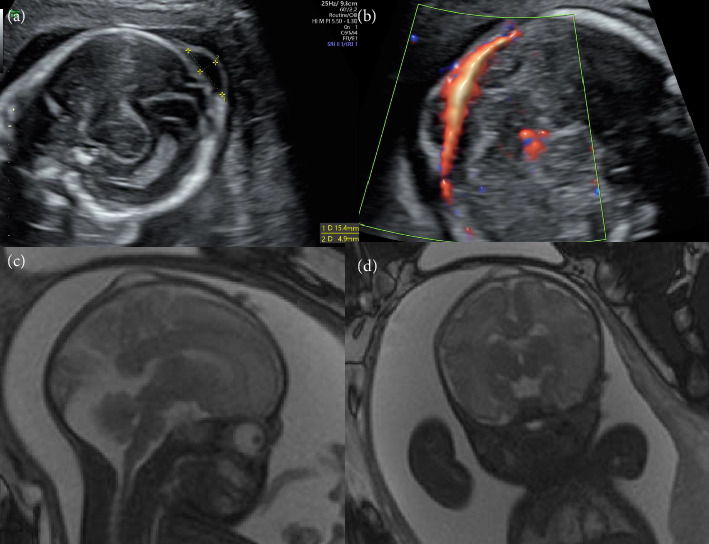
The cyst observed under the scalp of the fetus using two-dimensional ultrasonography and magnetic resonance imaging (MRI). (a) Image obtained using two-dimensional ultrasonography at 20 weeks of gestation. A 15 × 5 mm fetal scalp cyst and a vague border between the cyst and fetal skull bone can be seen. (b) Image obtained using two-dimensional ultrasonography with power Doppler at 22 weeks of gestation. No blood flow from the intracranial space to the scalp cyst could be observed; however, blood vessels were present. (c) Image of the lateral view obtained using T2-weighed MRI at 32 weeks of gestation. A border between the scalp cyst and fetal skull bone can be seen. (d) Image of the coronal view obtained using T2-weighed MRI at 32 weeks of gestation. A border between the scalp cyst and fetal skull bone can be seen.

A healthy 35-year-old Japanese woman (gravida 3, para 1) with no family history of congenital or genetic disorders was referred to our hospital after a small cyst was detected under the scalp of her fetus at 20 weeks and 4 days of gestation. She had conceived following in vitro fertilization, and had been prescribed levothyroxine 50 *μ*g per day for hypothyroidism. An ultrasonography examination revealed a single fetus with a 15 × 5 mm parietal cyst on the scalp that was not connected to the blood flow, and did not include the tissue of the brain parenchyma (Figures 1(a), 1(b)). Ventriculomegaly and other structural abnormalities were not observed. Based on these findings, we suspected a sinus pericranii or fetal epidermal cyst. Fetal MRI performed at 32 weeks and 4 days of gestation confirmed a fluid-filled mass 15-mm in diameter, and ruled out the connection to the intracranial space from the fetal scalp cyst (Figures 1(c), 1(d)). The size of the fetal scalp cyst was stable, and the growth of the fetus remained normal until birth.

**Figure 2:**
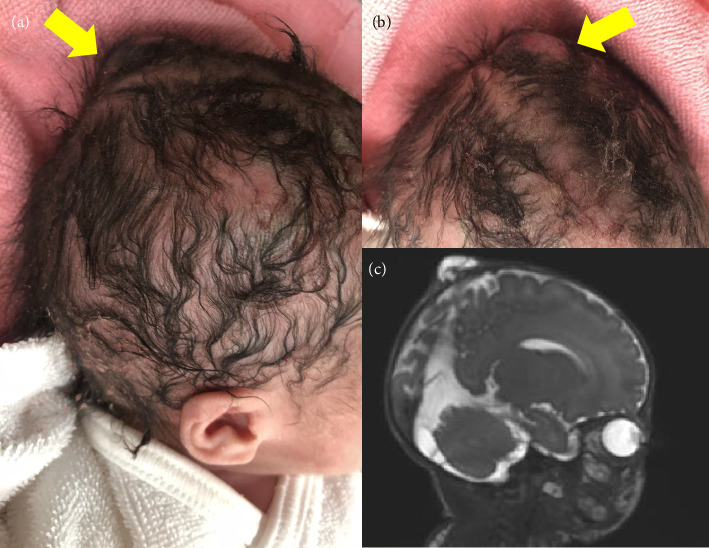
Gross findings and postnatal magnetic resonance imaging (MRI) of the scalp cyst. (a) Lateral view of the scalp cyst (yellow arrow). (b) Frontal view of the scalp cyst. A small lesion is observed (yellow arrow). (c) Lateral view of the small scalp cyst connected to the intracranial space observed using T2-weighed MRI.

**Figure 3:**
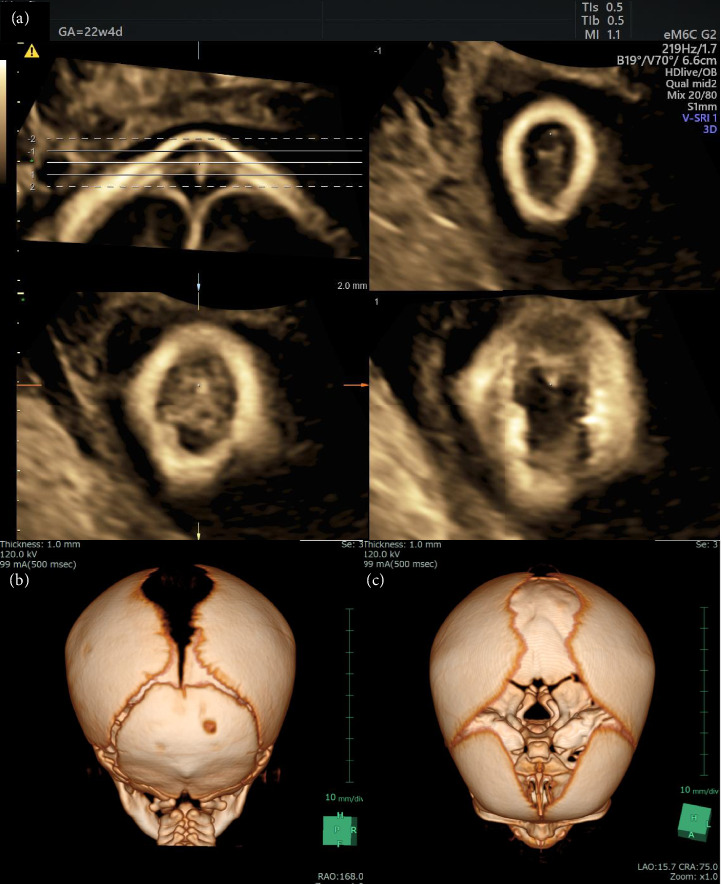
Images obtained during volume contrast imaging (VCI) of the head at 22 weeks of gestation and during postnatal computed tomography (CT). (a) The circular defect of the fetal skull bone, connecting to the sagittal suture in the right lower image, can be seen. (b) The posterior view of postnatal CT image showing the posterior fontanelle and skull bone defect of the neonate. (c) The parietal view of postnatal CT image showing the anterior fontanelle and skull bone defect of the neonate.

At 38 weeks and 2 days of gestation, a live female neonate with a weight of 2830 g was born via elective cesarean delivery because of the mother's history of cesarean delivery. The Apgar scores at 1 and 5 min were 8 and 9, respectively. A small bump on the head of the neonate was observed during the gross examination, but no other remarkable abnormalities were detected (Figures 2(a), 2(b)). However, MRI on postnatal day 5 revealed a small connection between the intracranial space and scalp cyst, resulting in a diagnosis of a parietal MC (Figure 2(c)). Subsequently, we reanalyzed the prenatal ultrasonography data obtained at 22 weeks of gestation using VCI, which revealed the circular defect of the fetal skull bone connecting to the sagittal suture (Figure 3(a)). Computed tomography (CT) on postnatal day 34 revealed a bone defect on the parietal skull (Figures 3(b), 3(c)). MC repair was performed on postnatal day 47. The postoperative course was uneventful, and the patient was discharged on postnatal day 53 without complications.

## 3. Discussion

Determining the correct diagnosis of small cysts under the scalp of a fetus is difficult [1–9]. As shown in Table 1, the diagnostic process should proceed based on the results of US and MRI findings. Although benign epidermal scalp cysts are generally not severe, they are associated with an increased risk of pregnancy termination [5]. Additionally, MC can have a critical effect on fetuses and their parents, as approximately 80% of MC cases result in pregnancy termination or intrauterine fetal demise [4, 10]. Therefore, although small MCs have a relatively benign prognosis, a precise prenatal diagnosis is important. Accurate prenatal diagnosis can help the parents of a fetus with a MC understand the prognosis and manage postnatal care. Fetal ultrasonography and MRI are very useful in the prenatal diagnosis of medium and large MCs; additionally, they are relatively easy to detect and can identify other neurological abnormalities, such as ventriculomegaly, Chiari malformation, and hydrocephalus [10]. However, detecting a clear border between the meninges and skull bone is difficult when using two-dimensional ultrasonography to evaluate small MCs [1, 4]. The bone has high echogenicity, and the meninges are slightly more echogenic than the tissue of the brain parenchyma. Detecting a clear border between the meninges and skull bone using two-dimensional ultrasonography was complicated in this case (Figures 1(a), 1(b)).

Performing three-dimensional power Doppler imaging with three-dimensional ultrasonography can differentiate vascular structures from intracranial spaces, thereby allowing the diagnosis of small MC under the scalp of the fetus [2]. An offline multiplanar analysis using three-dimensional ultrasonography allows the virtual navigation in all three orthogonal planes of the region of the cyst and the detection of small bone defects in cases without blood flow [1]. We could not detect blood flow from the intracranial space to the scalp cyst using ultrasonography with power Doppler in this case. Additionally, we mistakenly considered the cleavage to be the anterior fontanel or an ultrasound artifact when using only normal three-dimensional ultrasonography because the bone defect was very close to the anterior fontanelle on the head CT image obtained postnatally (Figure 3). These factors resulted in difficulty diagnosing the small MC. Initially, we considered a sinus pericranii or benign epidermal cyst as the diagnosis. Although fetal MRI did not reveal any connection between the intracranial space and small scalp cyst, VCI clearly displayed the bone defect. If VCI is used to determine the prenatal diagnosis, then MCs may be diagnosed more confidently, resulting in better prenatal counseling and fewer evaluations such as fetal MRI. This case suggests that the use of VCI with three-dimensional ultrasound data is helpful for determining the prenatal diagnosis of small fetal MCs, as this combination allows the detection of bone defects of the fetal head.

## Figures and Tables

**Table 1:** Ultrasound (US) and magnetic resonance imaging (MRI) findings for the differential diagnosis of fetal small scalp cysts.

Differential diagnosis of fetal small scalp cyst	US findings	MRI findings	Reference (s)
Meningocele	Homogenic or heterogenic low echoic lesion; small vessels crossing the skull observed with power Doppler; small skull defect assessed using three-dimensional US and volume contrast imaging analysis	Homogeneity and high signal intensity in a cyst on a T2-weighted image; connection between the intracranial space and a small scalp cyst; glial tissue within a cyst	[1, 2]
Epidermal scalp edema	Homogenic low echoic lesion	Homogeneity and high signal intensity in a cyst on a T2-weighted image; a lesion that does not extend intracranially	[3–5]
Sinus pericranii	Soft tissue mass in the cyst; skull defect; vein connecting the mass to the dural sinus	Soft tissue mass of the scalp with a skull bone defect on a T2-weighted image; vein connecting the mass to the superior sagittal sinus	[6]
Teratoma	Mixed low-to-high echoic lesion	No connection between the intracranial space and a cystic lesion	[7]
Lipoma	Mixed low-to-iso-echoic lesion	Homogeneity and similarity to subcutaneous fat intensity on a T1-weighted image	[8]
Hemangioma	Heterogenic mixed lesion; abundant blood flow signals within the mass with color Doppler; internal honeycomb echoes	Multiple tortuous vessels with low signal intensities on a T2-weighted image	[9]

## Data Availability

All available data are presented in the case report. There are no additional data available.
